# Enhanced Antidepressant Activity of Nanostructured Lipid Carriers Containing Levosulpiride in Behavioral Despair Tests in Mice

**DOI:** 10.3390/ph16091220

**Published:** 2023-08-29

**Authors:** Sadia Tabassam Arif, Muhammad Ayub Khan, Shahiq uz Zaman, Hafiz Shoaib Sarwar, Abida Raza, Muhammad Sarfraz, Yousef A. Bin Jardan, Muhammad Umair Amin, Muhammad Farhan Sohail

**Affiliations:** 1 Riphah Institute of Pharmaceutical Sciences, Riphah International University, Islamabad 44000, Pakistan; tabassamsadia96@yahoo.com (S.T.A.); mayubkhan73@yahoo.com (M.A.K.); shahiq.zaman@riphah.edu.pk (S.u.Z.); 2 Faculty of Pharmaceutical Sciences, University of Central Punjab, Lahore 54000, Pakistan; shoaib.sarwar@ucp.edu.pk; 3 Nanomedicine Research Laboratory, National Institute of Lasers and Optronics (NILOP), PIEAS, Islamabad 45650, Pakistan; abida_rao@yahoo.com; 4 College of Pharmacy, Al Ain University, Al Ain 64141, United Arab Emirates; muhammad.sarfraz@aau.ac.ae; 5 Department of Pharmaceutics, College of Pharmacy, King Saud University, Riyadh 11451, Saudi Arabia; ybinjardan@ksu.edu.sa; 6 Department of Pharmaceutics and Biopharmaceutics, University of Marburg, 35032 Marburg, Germany; umairami@staff.uni-marburg.de; 7 Riphah Institute of Pharmaceutical Sciences, Riphah International University Lahore Campus, Lahore 54000, Pakistan

**Keywords:** levosulpiride, antidepressant, nanostructured lipid carriers, acute toxicity, in vivo imaging

## Abstract

The potential of levosulpiride-loaded nanostructured lipid carriers (LSP-NLCs) for enhanced antidepressant and anxiolytic effects was evaluated in the current study. A forced swim test (FST) and tail suspension test (TST) were carried out to determine the antidepressant effect whereas anxiolytic activity was investigated using light–dark box and open field tests. Behavioral changes were evaluated in lipopolysaccharide-induced depressed animals. The access of LSP to the brain to produce therapeutic effects was estimated qualitatively by using fluorescently labeled LSP-NLCs. The distribution of LSP-NLCs was analyzed using ex vivo imaging of major organs after oral and intraperitoneal administration. Acute toxicity studies were carried out to assess the safety of LSP-NLCs in vivo. An improved antidepressant effect of LSP-NLCs on LPS-induced depression showed an increase in swimming time (237 ± 51 s) and struggling time (226 ± 15 s) with a reduction in floating (123 ± 51 s) and immobility time (134 ± 15 s) in FST and TST. The anxiolytic activity in the light–dark box and open field tests exhibited superiority over LSP dispersion. Near-infrared images of fluorescently labeled LSP-NLCs demonstrated the presence of coumarin dye in the brain after 1 h of administration. An acute toxicity study revealed no significant changes in organ-to-body weight ratio, serum biochemistry or tissue histology of major organs. It can be concluded that nanostructured lipid carriers can efficiently deliver LSP to the brain for improved therapeutic efficacy.

## 1. Introduction

Mental and behavioral disorders affect approximately one billion people globally, as reported by the World Health Organization (WHO). According to an estimate, 12 billion workdays are lost every year because of depression and anxiety, which costs the global economy USD 1 trillion [1]. Among these psychiatric diseases, depression is the second highest threatening mental health illness, resulting in an increased economic burden worldwide [2]. Depression and anxiety are comorbid conditions with prominent features that include lack of motivation, emotional instability, sleep distress, loss of interest in social activities and a higher incidence of suicidal tendencies [3]. Disturbance in transmission from presynaptic to postsynaptic neurons of major neurotransmitters like dopamine, serotonin and norepinephrine is the leading cause of depression [4]. The therapeutic response of depressed, anxious patients to available treatments is disappointing despite the adherence. The continuous presence of antidepressants at the site of action for a prolonged period is necessary for their efficacy. Route of administration plays a pivotal role in delivering therapeutically effective drugs to the brain. The blood-brain barrier, efflux transporters and blood-cerebrospinal fluid barrier portray major hindrances for orally administered drugs circulating in the blood after absorption [5].

An efficient drug delivery system that can maximize drug concentration in the brain should possess prominent features of prolonged-release rate, improved bioavailability and enhanced permeability [6]. The administration of antidepressant drugs through lipid nanocarriers satisfies these assumptions [7]. Nanostructured lipid carriers (NLCs) purport to be a promising formulation to deliver lipophilic drugs to the brain. Being a lipid-based formulation, it has the advantage of bioacceptability and rapid uptake by the brain. Nanostructured lipid carriers are the second generation of lipid nanoparticles that overcome the limitation of other lipid formulations including larger particle size, instability, drug expulsion, inability to target brain tissues and problems in scaling up [8]. These lipid nanoparticles have improved the transport of drugs to the brain by offering high drug entrapment, improved biological physical stability, reduced toxicity, and loading of both hydrophilic and lipophilic drugs [9,10]. The imperfections created by liquid lipids prevent the polymorphic transitions of solid lipids to a highly ordered crystal structure leading to the conception of NLCs which are solid, but not crystalline. This is derived from the fact that the crystallization process itself causes the expulsion of the drug, as evident in first generation counterparts composed of solid lipid alone [11]. By using binary mixtures of solid lipids and liquid lipids, the particles become solid after cooling but do not crystallize. NLC has easily stabilized with the minimum possible concentration of surfactants along with the best results of stability, entrapment, and release [12].

**Figure 1:**
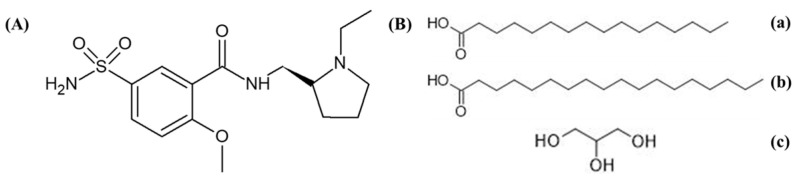
Chemical structure depiction of (**A**) levosulpiride and (**B**) component compounds of Precirol ATO 5. (a) Palmitic Acid, (b) Stearic Acid and (c) Glycerin.

Levosulpiride (Figure 1A) is a benzamide derivative with antipsychotic, antidepressant and prokinetic activity. It inhibits the dopamine D_2_ receptors at the trigger zone in the central and peripheral nervous system [13]. Due to the high selectivity of D_2_ inhibition, LSP has a low sedation and hypotensive effect. Levosulpiride has low bioavailability (20–30%) at the dosage range of 100–200 mg after oral administration. The time to reach peak plasma concentration is 3 h with a half-life of approximately 6 h depending on the dose and route of administration. Being less soluble with low permeability, levosulpiride has difficulty penetrating the blood-brain barrier after oral administration [13].

In a previous study, we developed, optimized and characterized LSP-loaded NLCs which significantly increased bioavailability and enhanced the prokinetic efficacy of LSP after oral administration, but the antipsychotic and antidepressant effect of LSP-NLCs was not determined. Our current study aims to investigate the antipsychotic and antidepressant efficacy of LSP when administered through NLCs. Near-infrared fluorescence (NIRF) imaging was performed to determine the biodistribution of LSP-NLCs after administration. An acute toxicity study of LSP was carried out in rats.

## 2. Results and Discussion

### 2.1. Particle Characterization and Entrapment Efficiency

**Figure 2:**
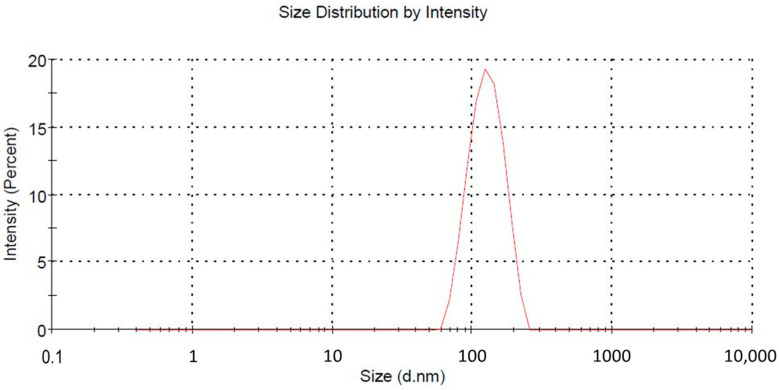
Particle size distribution curve.

The developed optimized LSP-NLCs were subjected to particle size analysis, and PDI value and zeta potential were also measured. Optimized LSP-NLCs showed a mean particle size of 151.2 ± 1.06 nm in terms of % intensity (Figure 2). The obtained PDI value of LSP-NLCs was 0.25 ± 0.02, showing a narrow size and uniformly homogeneous distribution. The literature suggests that nanoparticles with a size of less than 200 nm show efficient absorption in the intestine and have a tendency to bypass uptake by the reticuloendothelial system and hepatocytes. These characteristics anticipate an enhanced LSP bioavailability with extended blood circulation and effective transport of LSP-NLCs to the brain to attain the desired therapeutic levels [14,15]. LSP-NLCs showed a zeta potential value of −23.17 ± 3.37 mV. Previously, it was reported that Tween 80 and Labrasol were responsible for the steric stabilization of the nano-formulation. In the presence of steric stabilization along with electrostatic stabilization, the lipid nano-formulations exhibited adequate physical stability at a zeta potential value of −20 mV. A zeta potential value of −23.17 ± 3.37 mV on the surface of LSP-NLCs demonstrated the LSP orientation within the lipid matrix and the prospects for physical stability of LSP-NLC formulation. Moreover, the highly negatively charged nanoparticles were reported to be more favorable for brain drug delivery than positively charged nanoparticles as they may compromise the blood-brain barrier integrity [16,17]. The LSP-NLCs showed a high incorporation efficiency of 86 ± 3.1%, which could be due to the addition of the liquid lipid in the lipid matrix. 

### 2.2. Biodistribution Study

**Figure 3:**
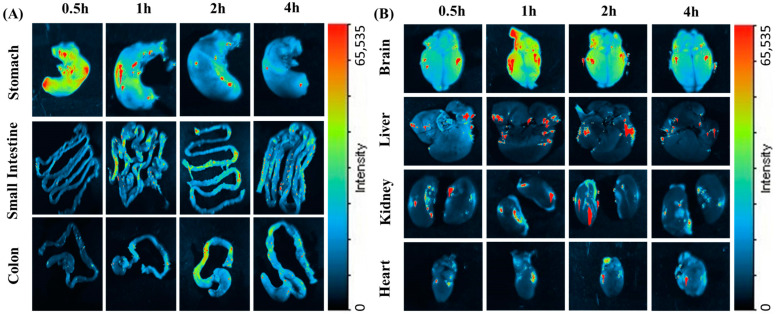
Fluorescent images of excised mice organs (**A**) stomach, small intestine and colon after oral administration (**B**) brain, liver, kidneys and heart after I.P injection of coumarin-6 labeled NLCs.

LSP-NLCs were physically tagged with coumarin 6 to make them fluorescent to track the biodistribution after subjecting them to NIRF imaging. The movement of F-NLCs in the GIT was tracked by harvesting the stomach, intestine and colon after oral administration at 0.5 h, 1 h, 2 h and 4 h. Ex vivo imaging studies were examined in excised organs (brain, heart, liver, kidneys) to identify the relative fluorescent signal at the abovementioned time points after an I.P. injection. The reason for the selection of the I.P route to determine the biodistribution of LSP-NLCs in different organs other than the GIT system was to avoid the digestion of NLCs in the small intestine as the coumarin-6 was physically tagged within the F-NLCs. The results in Figure 3A show ex vivo optical imaging in GIT to confirm the F-NLC movement. At the initial time point (0.5 h), a strong fluorescent signal was observed in the stomach which decreased with the increasing time intervals showing the gastric emptying of the stomach. Subsequently, the fluorescent signal in the initial part of the intestine was observed at the time point of 1 h after oral administration. A drop in florescent intensity with the movement to the lower portion of the intestine was translated as the absorption of F-NLCs in the intestine and GIT movements [18]. The fluorescence that appeared in the colon after 2 h was consistent throughout the colon and disappeared after 4 h, demonstrating no absorption in the colon. It is assumed that the florescent dye was disassociated from F-NLCs after the digestion process in the intestine and the luminal content was a mixture of unabsorbed coumarin and lipid digests. The ex vivo images exhibited in Figure 3B show that the signal for fluorescence intensity was increased in a time-dependent manner in the obtained organs, which indicates that the F-NLCs were absorbed from the peritoneal cavity and were available in the blood circulation from which they were distributed to different organs. The brain was the main organ of interest to study the biodistribution of NLCs as the concentration of LSP in the brain determines the therapeutic efficacy of the LSP in depression and anxiety treatments along with the role of NLCs in brain drug delivery. The fluorescent images of the brain showed a low intensity of fluorescence after 30 min of I.P injection whereas the maximum brain accumulation was observed after 1 h. Afterward, a decrease in fluorescence was detected. The strong fluorescence signal in the brain established the fact that LSP-NLCs have the tendency to pass the blood-brain barrier and increase the bioavailability of LSP in the brain. The possible mechanisms behind this phenomenon could be smaller particle size, which enhances permeability due to lipid nature [19,20], membrane fluidization due to the presence of surfactants, efflux pumps blocking the brain endothelial cells [21,22], and possible endocytosis or cytopempsis of the NLCs [23]. Furthermore, previously it was reported that low molecular weight and lipophilicity of the drug also play a role in the passive diffusion of the drug molecule which could also be a factor in enhanced brain delivery of LSP. Fewer signals in the liver and kidneys showed little accumulation of F-NLCs in the liver and the kidneys. The ex vivo images of the heart also presented a faint signal showing an insufficient amount of F-NLCs [13,24].

### 2.3. Antidepressant and Anxiolytic Activity

**Figure 4:**
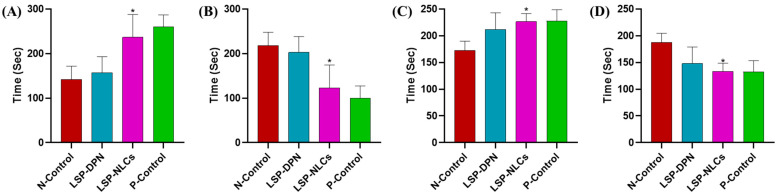
The effect of LSP dispersion (LSP-DPN) and LSP-NLCs on the (**A**) swimming time, (**B**) floating time in the forced swim test, (**C**) struggle time and (**D**) immobility time in the tail suspension test in LPS-induced depressed mice. The data are presented as mean ± S.D (n = 3). Data were analyzed by one-way ANOVA followed by Dunnett’s test. * *p* ˂ 0.05 compared to the negative control (LPS only) group.

In an attempt to elucidate the treatment response to underlying psychiatric illness, complementary behavioral tests were performed to assess LPS-induced antidepressant and anxiolytic effects. LPS has been extensively used to induce behavioral changes in rodents which mimic depression and anxiety symptoms. These symptoms exhibit as behavioral despair, disturbance in the expression of inflammatory markers and alteration in neurotropic transmission. The antidepressant and anxiolytic effect of LSP-NLCs and LSP-DPN was evaluated in an LPS-induced depression and anxiety model after oral delivery. Struggling and immobility time in the forced swim test and tail suspension test were taken as a measure of the efficacy of the optimized LSP-NLCs. The LPS injection significantly reduced the swimming time of mice (142 ± 30 s) in the negative control group to which no treatment was given, indicating the induction of depression as compared to the P-Control group (260 ± 27 s). When LSP-NLCs and LSP dispersion were administered orally there was increased swimming time (237 ± 51 s & 157 ± 36 s) and reduced immobility (123 ± 51 s & 203 ± 35.6 s) as compared to the N-Control group (Figure 4A,B). The tail suspension test showed similar results to those from the FST. The obtained results (Figure 4C,D) suggest that LSP-NLCs substantially increased the struggling time to 226 ± 15 s and reduced immobility time to 134 ± 15 s, which was greater than that of LSP-DPN administered orally (212 ± 31, 148 ± 30.8 s). The results mentioned in Table 1 show a significant increase (119 ± 10 s) in the time spent and number of entries (7 ± 2) in the lightbox as compared to LPS-induced anxious mice. Similarly, the mouse group treated with LSP-NLCs showed an increase in the time spent in the central compartment and the number of compartments crossed (Table 1). Results from the pharmacodynamics studies (behavioral despair) revealed the effective delivery of levosulpiride loaded in NLCs through the oral pathway. Levosulpiride delivery in the form of NLCs enhanced the bioavailability of the LSP in systemic circulation ultimately leading to augmented brain delivery when compared with an equivalent dose of dispersion. The behavioral changes revealed in the pharmacodynamics studies in mice can be explained by the antagonist effect of LSP on D_2_ receptors. The selective blockade of the D_2_ receptor at presynaptic and postsynaptic neurons increases the behavioral response in depressed and anxious mice by controlling the synthesis and release of dopamine [25,26].

### 2.4. Acute Oral Toxicity Study

#### 2.4.1. Body Weight Measurements, Observation of Clinical Signs and Food Consumption

**Figure 5:**
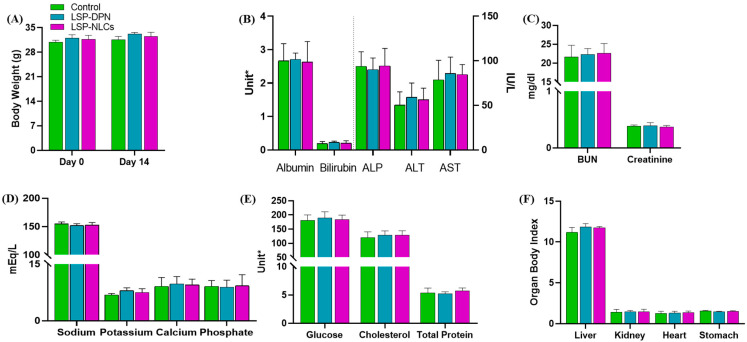
Evaluation of acute oral toxicity through analysis of (**A**) body weight comparison, serum biochemical analysis of blood, (**B**) liver function tests, (**C**) renal function tests, (**D**) serum electrolytes, (**E**) glucose, cholesterol, total protein and (**F**) organ body index after treatment with LSP dispersion (LSP-DPN) and LSP-NLCs. Data are represented as mean ± S.D (n = 3). Unit = Albumin (g/dL), Bilirubin (mg/dL), ALP (IU/L), ALT (IU/L), AST (IU/L), Glucose (mg/dL), Cholesterol (mg/dL), Total protein (g/dL).

In vivo toxic effects of LSP and LSP-NLCs were evaluated at a comparatively higher concentration of 50 mg/kg of LSP. The treatments were orally administered to each mouse in the treated groups whereas normal saline was given to the control group. After the administration of specific treatment, the mice were monitored for 48 h for specific parameters (any change in the behavior pattern, physical appearance, occurrence of clinical or toxicological symptoms, feed consumption, and mortality) and after that were observed daily to check the occurrence of any toxic event macroscopically. No animal mortality or prominent exterior signs of toxicity were recorded. All the experimental animals showed normal patterns of physical condition and behavior. The body weights of the animals at day 0 and day 14 are given in Figure 5A. No significant change in body weight among the control and treated groups was noted throughout the duration of the study. On day 14, before the utilization of the mice to collect different organs for further study, the blood samples were taken from all mice individually and hematology and serum biochemistry analyses were performed.

#### 2.4.2. Hematology Analysis

For a drug nanocarrier system, the most critical task is to ensure compatibility with the blood to avoid any unwanted inflammatory response which mostly occurs due to incompatibility with blood components. The hematological data results in Table 2 exhibit that no statistically significant differences were present among the treatment-exposed groups and the control group. The complete blood count results of all treated mice showed normal levels of total red blood cells, white blood cells, hemoglobin, and platelet count as well as packed cell volume, hematocrit, mean corpuscular volume and mean corpuscular hemoglobin in the peripheral blood and the results of treated groups remained in close proximity to the results for the control group.

#### 2.4.3. Serum Biochemistry

Serum biochemistry parameters were analyzed to check for any abnormal effect of the LSP suspension and LSP-NLCs on the integrity and functions of the liver and kidneys. Liver function test parameters are shown in Figure 5B. The functionality of liver cells is described by the albumin levels which were not affected by the LSP dispersion or LSP-NLCs treatment. An insignificant difference in the level of alanine aminotransferase and alkaline phosphate was observed with LSP-DPN and LSP-NLCs compared to that of the controls and the values fall within the acceptable limits. Cellular integrity of the liver cells is portrayed through alanine aminotransferase and alkaline phosphate which are produced as indicators of the function of liver cells. An increased alanine aminotransferase level specifies cell damage or necrosis due to the outflow of this enzyme from liver cells to the blood. Elevated alkaline phosphate levels also illustrate cholestatic liver disease with liver cell damage connected with the bile duct. Normal aspartate aminotransferase values of the treated groups with an insignificant difference from the control show the integrity of the vital organs. A high aspartate aminotransferase level is a sign of organ damage. The liver is also the main production site for serum proteins and any variation in the total protein content shows liver abnormality. No significant changes were observed in the total bilirubin level. However, the total protein levels were unaffected by LSP-DPN and LSP-NLC treatment. The influence of LSP-NLCs on liver function tests was insignificant (*p* > 0.05). To evaluate the consequence of LSP-NLC treatment on the kidneys, renal function tests (RFTs) were performed. The outcomes in Figure 5C disclose no significant variation from the RFTs reference values among the treated and untreated control groups. All the treatments did not affect the creatinine and BUN levels. For further toxicity assessment induced by LSP-NLC treatment, serum electrolytes (Na, K, Ca, and P) were assessed. The results (Figure 5D) revealed no changes in serum Na and K levels in the case of LSP-NLCs compared to the control. No changes were observed in the levels of Ca and P compared with the control. The results in Figure 5E present no alteration in glucose levels and cholesterol levels were also unchanged in all groups compared to the control [27,28].

#### 2.4.4. Organ to Body Ratio

The weights of the vital organs are considered a good parameter for the evaluation of in vivo toxicity. After 14 days of treatment exposure, the organ-to-body ratio of the vital organs including the liver, kidneys, heart and stomach was calculated. The organ-to-body index results (Figure 5F) exhibited no significant changes for all test organs from the treated groups when compared to the control.

#### 2.4.5. Histopathology of Vital Organs

**Figure 6:**
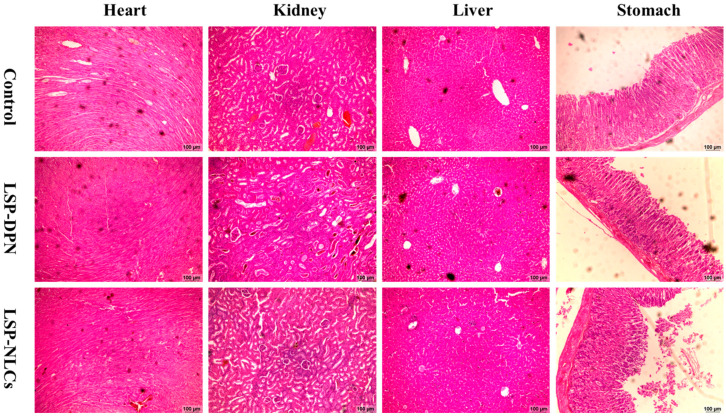
Histological microscopic images of vital organs of heart, kidneys, liver and stomach of mice on day 14 after treatment with LSP dispersion (LSP-DPN) and LSP-NLCs.

The macroscopic inspection of the heart, kidneys, liver and stomach did not display any observable variations or lesions on these organs. For further investigation, tissue histology studies were executed on the slides prepared from test organs, as presented in Figure 6. The normal morphological architecture was observed in microphotographs of tissue slides of all the vital organs from the control and treated groups. The macroscopic inspection of the heart, kidneys, liver and stomach did not display any observable variations or lesions on these organs. A normal arrangement of cardiac myofibrils and myocytes was observed without any structural change. Also, no signs of cardiac damage, necrosis, infiltration, inflammation, hemorrhage, vacuolization, or myocardial infarction were observed in all treated groups. Figure 6 shows that no morphological change occurred in the kidneys of the treated groups. The glomerular structure presented a regular appearance in the treated groups, which was similar to the control groups, without any interstitial and intraglomerular congestion or tubular atrophies. All the nephrons appeared to be normal without any degeneration or necrosis. In the microscopic examination of the liver slides, the hepatocytes displayed normal morphology with no signs of fatty accumulation, injury, or necrosis. These findings support the outcomes obtained from liver function tests and renal function tests demonstrating the safety of LSP-NLCs. Similarly, no signs of disruption were seen in the microscopic images of the stomach among the control and treated groups. The gastric mucosa appeared to be intact with a typical histological architecture and normal epithelium and glands.

## 3. Materials and Methods

### 3.1. Materials

Levosulpiride was a kind gift from Bio-Lab Pvt. Ltd., Islamabad, Pakistan. Precirol^®^ ATO5 (Glyceryl palmitostearate, Figure 1B) and Labrasol^®^ (Caprylocaproyl Polyoxyl-8 glycerides) were generous gift by Gattefossé (Cedex, France). Coumarin, Tween 80 (polyoxyethylenesorbitan monooleate) and Span 80 (Sorbitan Monooleate) were purchased from Sigma-Aldrich (St. Louis, MO, USA). Methanol, acetonitrile and chloroform of HPLC grade were purchased from Merck (Hohenbrunn, Germany). All other chemicals were of analytical grade and were used without further purification.

### 3.2. Preparation of LSP-NLCs

LSP-NLCs were prepared using a hot homogenization ultrasonication technique. For this purpose, solid and liquid lipids and Span 80 were weighed and put into a glass vial. To obtain lipid melt, the glass vial was maintained at 70 °C in a water bath. Then, a definite amount of LSP was added to the melted lipids and mixed well in the molten lipids to completely dissolve the drug. For the preparation of an aqueous phase, surfactant Tween 80 was dissolved in distilled water in a separate vial and heated up to 70 °C. Then, this hot aqueous phase was poured into the lipid phase and homogenized using a homogenizer (HG-15D, DAIHAN Scientific, Wonju, Republic of Korea at 15,000 rpm for 5 min. The temperature of the system was maintained at 70 °C. A coarse o/w emulsion was obtained which was further sonicated for 3 min at an amplitude of 50% and power of 100 W using a probe sonicator (Vibracell™ VCX750; Sonics and Materials Inc., Newtown, CT, USA). The obtained nano-emulsion was rapidly cooled down in a glass jar filled with ice to obtain LSP-NLCs.

In our previous study, an initial screening was carried out to select the suitable solid, liquid lipid and surfactant. Precirol ATO 5 was selected as the solid lipid and Labrasol was chosen as the liquid lipid. The preferred surfactants used were Span 80 and Tween 80. The selection of the lipids was based on the solubility of levosulpiride in different lipids, whereas the selection of the surfactants was determined by the homogeneity and stability of the formulation. The LSP-loaded NLCs were optimized based on particle size, PDI value and entrapment efficiency. Design-Expert software (version 13, Stat-Ease Inc., Minneapolis, MN, USA) was used to find the optimal mixture composition of lipids and surfactants. Numerical optimization with desirability function was carried out to obtain the optimal formulation. The composition of the optimized formulation was 80.55% Precirol^®®^ ATO 5, 19.45% Labrasol and 5% Tween 80/Span 80 mixture. The detailed results of the preliminary study have been described in Sadia et al. [29].

### 3.3. Particle Size, PDI and Zeta Potential Measurement

The mean particle size and PDI value for optimized LSP-NLCs were determined by a dynamic light scattering technique using a Zetasizer Nano ZSP (Malvern Instruments, Worcestershire, UK), whereas the zeta potential was measured using a phase analysis light scattering approach. The formulation was properly diluted with ultrapure water before measurements were taken and the analysis was carried out in triplicates [30].

### 3.4. Entrapment Efficiency

The entrapment efficiency (*EE*) of LSP-NLCs was calculated according to Equation (1). The untrapped drug and free lipids were separated by centrifugation of 1 mL of the LSP-NLCs formulation at 4 °C and 12,000 rpm for 15 min. The obtained LSP-NLCs were reconstituted with 1 mL of methanol and the concentration of the LSP incorporated in the NLCs was determined spectrophotometrically at 237 nm.

(1) $$EE\;\left(\%\right)=\frac{Amount\;of\;LSP\;in\;NLCs}{Total\;amount\;of\;LSP\;added}\times 100\;\;\;$$

### 3.5. In Vivo Studies

#### Experimental Animals

Assessment of the antidepressant and anxiolytic efficacy of LSP-NLCs via biodistribution and acute toxicity studies was carried out on albino mice (30 ± 5 g). All the experimental animals were obtained from the animal house of Riphah International University, Islamabad Pakistan. To acclimatize the animals to the lab environment, standard conditions were maintained. All animal studies were in accordance with the National Institute of Health policies, in line with the animal welfare act, and approved by the Research and Ethics Committee of Riphah Institute of Pharmaceutical Sciences (Approval# REC/RIPS/2019/015). 

### 3.6. Biodistribution Study

The biodistribution of LSP through NLCs was carried out by performing a qualitative analysis using fluorescent-labeled NLCs (F-NLCs). F-NLCs were developed by mixing coumarin-6 (0.5% *w*/*w*) in the lipid melt during the preparation of NLCs before the addition of the aqueous phase. To separate the F-NLCs from the unentrapped coumarine-6 and free lipids, centrifugation was performed at 12,000 rpm for 15 min. In order to determine the fate of LSP-NLCs in the gastrointestinal tract (GIT), F-NLCs were given orally and to examine the LSP uptake in the brain and different organs, an intraperitoneal (I.P) injection equivalent to the oral dose was administered. Twenty-four male albino mice were divided into eight groups, four groups for the oral route and four groups for the I.P route (one group for each time point). Ex vivo imaging of different organs collected from sacrificed mice was performed at the predetermined time intervals of 0.5 h, 1 h, 2 h and 4 h after administration. Fluorescence imaging was performed using I-Box Explorer^2^ (iBox^®^ Explorer^2^ Imaging Microscope, UVP Ltd., Cambridge, UK) equipped with an automated BioLite™ Multi-Spectral Light Source, and the system excitation and emission filter were set at 535/45 and 605/50, respectively. A 3.2 MP OptiChemi 695 camera was used to capture the images and the images were captured at 0.17× magnification. The recorded images were interpreted using Vision Works^®^ LS Acquisition software [18,31].

### 3.7. Anti-Depressant and Anxiolytic Activity

**Figure 7:**
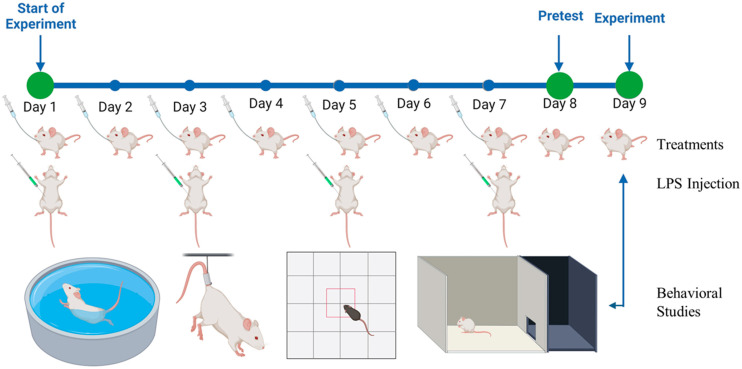
The pictorial illustration of LPS-induced depression and anxiety model (created with BioRender.com, accessed on 23 July 2023).

The antidepressant and anxiolytic activity of LSP-loaded NLCs was investigated using a lipopolysaccharide (LPS)-induced depression and anxiety model in mice. Twelve experimental animals were divided into four groups (three mice in each group). Group-1 served as a positive control (P-Control) and was administered with 0.5 mL normal saline daily whereas group-2 was selected as negative control (N-Control) and was given LPS only to induce depression and anxiety. Group-3 and group-4 were treated groups and received individual LSP-DPN and LSP-NLCs daily at a dose equivalent to 5 mg/kg of LSP. Water was used as a vehicle to disperse the free LSP. The N-control group and treated groups also received intraperitoneal injections of LPS (500 µg/kg) on alternate days for 7 days. A pretest was conducted on day 8 of treatment followed by actual behavioral tests on day 9. The pictorial illustration of the LPS-induced depression and anxiety model is shown in Figure 7. 

#### 3.7.1. Behavioral Studies

To analyze the antidepressant activity of the LSP-NLCs, forced swim and tail suspension tests were carried out. Whereas, light–dark box and open field tests were performed to evaluate the anxiolytic behavior.

##### Forced Swim Test

The forced swim test, known as Porsolt’s test, was carried out by gently placing the mice in a transparent glass cylinder measuring 50 × 21 × 21 cm, filled with water up to 35 cm. The water was maintained at room temperature 25 + 2 °C and replaced with fresh water after each test to avoid the influence of contaminated water. The struggling motions of mice were recorded for 6 min. The struggling and immobility times were recorded for all mice. Immobility was considered as a loss of struggling effort to escape the container. Swimming time was registered as a measure of mobility. Swimming was considered when forelimbs or hindlimbs moved in a paddling manner. The mice were removed from the water at the end of the experiment, dried with a towel and placed in a separate cage [6].

##### Tail Suspension Test

The tail suspension test was conducted by suspending the mice at a height of 50 cm by their tails and the tails were held away from the body with climb stoppers. Struggling and immobility time was videotaped for 6 min. Immobility was measured by the absence of any movements [32].

##### Light and Dark Box Test

For the light and dark experiment, a rectangular wooden box with dimensions of 44 × 21 × 21 cm, divided into two compartments separated by a wooden board with an opening in its base for the movement of mice from one to another compartment, was used. The larger light compartment was painted white in color and lit up with a bulb and the smaller compartment was painted black. Each mouse was placed in the light compartment and the time it spent in each compartment and the number of entries were recorded for 6 min [33].

##### Open Field Test

The open field test was used to determine the effect of LSP-NLCs on the locomotor activity of the mice. For this purpose, the mice were separately positioned in the center of a box (40 × 60 × 50 cm) to which they were not habituated before the test. The locomotor activity of the animals was observed immediately up to 6 min. Time spent in the central compartment and the number of line crossings were the parameters considered for the activity. The apparatus was cleaned after each mouse activity [34].

### 3.8. Acute Oral Toxicity Study

The acute oral toxicity of LSP and LSP-NLCs in mice was evaluated after a single oral dose for a period of 14 days. Mice were divided into 3 groups (n = 3) and retained under accustomed conditions of food and water. Group-1 was given LSP dispersion, group-2 was given LSP-NLCs, and group-3 served as control and was given normal saline. A dose of (50 mg/kg) was given orally through gavage [27].

#### 3.8.1. Body Weight Measurements and Observation of Clinical Signs and Food Consumption

The body weight of all the mice was measured on day 1 before dosing and after treatment on day 14. The experimental animals were visually observed initially for 48 h and then daily for 14 days for any change in behavior pattern, physical appearance, occurrence of clinical or toxicological symptoms, feed consumption, and mortality [35].

#### 3.8.2. Hematology Analysis

On day 14, blood samples of mice were collected from tail veins in EDTA vacuumed blood collection tubes and were analyzed by an automatic hematology analyzer (Sysmex KX-21, Sysmex Corporation, Kobe, Japan) for red blood cell counts (RBCs), total white blood cells (WBCs), hemoglobin (Hb) levels and total number of platelets in each sample. Packed cell volume (PCV) was measured manually by using capillary microhematocrit tubes [27,36].

#### 3.8.3. Serum Biochemistry

The serum was separated from the blood samples collected from tail veins and kept at −80 °C until further analysis. The renal and liver function test parameters and the concentration of serum enzymes, bilirubin, proteins and electrolytes were determined by using a semi-automated biochemistry analyzer (HumaLyzer 3500, Human Diagnostics, Wiesbaden, Germany) [28,37].

#### 3.8.4. Organ to Body Ratio

Upon completion of the study duration, the mice were euthanized and the heart, kidneys, liver and stomach were removed. After washing, the weight of all the organs was measured individually and treated groups were equated with the organ weight of the control group. Organ-body weight index was calculated by using the formula below [38].

Organ body weight index = Organ weight (g)/Body weight (g) ×100

#### 3.8.5. Histopathology of Vital Organs

A macroscopic examination of all the previously removed and washed organs was carried out to check for any abnormality or lesion formation against the control. The tissue histology slides of the heart, kidneys, liver and stomach were cautiously prepared by fixing the organs in paraffin and cross-sections of tissues of 0.5 μm in size were carefully cut using a microtome. The tissue sections were fixed onto glass slides, stained with H&E stain and examined microscopically (Olympus CX43) for structural changes and signs of toxicity [39,40].

### 3.9. Statistical Analysis

GraphPad Prism 8 software (version 8.0.2, San Diego, CA, USA) was used for statistical analysis of the data and results were presented as mean ± S.D. Statistical significance between the treated groups and controls was determined by comparing their mean values using one-way ANOVA followed by Dunnett’s test. The significance level was *p* < 0.05.

## 4. Conclusions

In this study, the antidepressant and anxiolytic performance of LSP-NLCs was investigated in vivo to reflect their therapeutic potential. LSP-NLCs significantly reduced depressive behavior in the animal model. Furthermore, F-NLCs show enhanced accessibility to brain tissues after intraperitoneal injection in biodistribution studies using ex vivo imaging analysis. However, LSP-NLCs were found to be safe after in vivo administration at higher doses and showed no signs of toxicity. These results suggest that LSP-NLCs could be a promising carrier system for the effective delivery of LSP to the brain for the treatment of depression.

## Figures and Tables

**Table 1:** Comparison of the results of light–dark box model (time spent and number of entries into lightbox) and open field test (number of quadrants invaded and central compartment resting time) among the different treated and control groups.

Groups	Light Dark Box Model		Open Field Test	
	Time Spent in Light Box	Number of Entries in the Lightbox	Number of Quadrant Invasion	Central Compartment Resting Time
Positive control	144 ± 73	5 ± 2	76 ± 19	11 ± 9
Negative control	52 ± 34	2 ± 2	19 ± 5	2 ± 2
LSP dispersion	76 ± 32	4 ± 1	44 ± 12	3 ± 2
LSP-NLCs	119 ± 10 *	7 ± 2 *	75 ± 22 **	6 ± 2 *

Data are presented as mean ± S.D (n = 3). Data were analyzed by one-way ANOVA followed by Dunnett’s test. * *p* < 0.05, ** *p* < 0.01 among treated vs. negative control.

**Table 2:** Hematological analysis of mice blood in acute toxicity studies after 14 days of oral administration of LSP dispersion (LSP-DPN) and LSP-NLCs.

Group	WBC	RBC	HCT	Hb	MCV	MCH	PCV	Platelets
	(×10^9^/L)	(×10^12^/L)	%	(g/dL)	(fL)	(pg)	(%)	(×10^9^/L)
Control	8.7 ± 2.3	8.4 ± 2.5	39.0 ± 2.9	14.5 ± 1.6	47.2 ± 3.1	14.4 ± 2.7	50.4 ± 7.4	762.3 ± 30.6
LSP-DPN	9.2 ± 2.5	9.4 ± 3.6	42.0 ± 2.5	15.1 ± 2.9	49.2 ± 1.9	15.0 ± 2.8	48.2 ± 5.1	755.1 ± 41.0
LSP-NLCs	8.9 ± 2.9	8.6 ± 2.8	40.3 ± 3.0	15.2 ± 2.2	48.8 ± 3.7	16.1 ± 2.4	48.8 ± 5.5	752.9 ± 52.5

Data are presented as mean ± S.D (n = 3).

## Data Availability

Data is contained within the article.
